# PalliPA: How can general practices support caregivers of patients at their end of life in a home-care setting? A study protocol

**DOI:** 10.1186/1756-0500-5-233

**Published:** 2012-05-14

**Authors:** Katja Hermann, Regine Boelter, Peter Engeser, Joachim Szecsenyi, Stephen M Campbell, Frank Peters-Klimm

**Affiliations:** 1 Department of General Practice and Health Services Research, University Hospital Heidelberg, Vossstr. 2, 69115, Heidelberg, Germany; 2 Health Sciences Research Group-Primary Care, University of Manchester, 7th Floor: Williamson Building, Manchester, M13 9PL, UK

**Keywords:** End-of-life care, Family caregivers, General practice, Participatory action research

## Abstract

**Background:**

The care of patients with a life-threatening, progressive and far advanced illness in a home-care setting requires appropriate individual care and requires the active support of family caregivers. General practice teams are usually the primary care givers and first contact and are best placed to offer support to family caregivers and to recognise and respond to the burden of care giving on family members. The aim of this project is to develop a best practice model for engaging with and supporting family caregivers.

**Findings:**

The project is framed as an exploratory trial for a subsequent implementation study, covering phases 0, I and II of the MRC (Medical Research Council) framework for development, design and evaluation of complex interventions. The project is a multi-method procedure and has two phases. In the first phase, which has already been completed, we used a reflective practice procedure where general practice teams were asked about how they currently deal with family caregivers. In the second phase, a participatory action research approach aims to improve identification and response to when support is necessary for family caregivers. Ten participating general practice teams each enrol 40 eligible patients and their family caregiver, to identify structures and tools feasible for use in their practice. Standardised self-reported questionnaires (Burden Scale for Family Caregivers and Quality of Life Questionnaire Core 15 Palliative) are being applied at study inclusion (prior to or during the implementation period) and after 6 and 12 months to explore implementation effects. Qualitative assessment of general practice teams’ experiences will be triangulated with the quantitative evaluation of the implementation.

**Discussion:**

This two-step approach, which is appropriate to primary palliative care in the German health care context, will enable general practice teams to develop feasible, acceptable and successful strategies for the implementation of best practice to successfully support family caregivers of patients at the end of life.

## Findings

### Background

The care of patients with a life-threatening, progressive and end of life illness in a home-care setting requires appropriate individual patient care and the active support of family caregivers. In this situation, general practitioners and practice staff are often the first and continuous health provider contact for family caregivers. The general practice team (GPT) therefore plays a pivotal role in the care of patients at the end of life and in supporting the patients’ family caregivers. GPTs are not only required to address the medical needs of patients, but also to recognise and respond appropriately to the physical and psychosocial burden of family caregivers [1], minimize unnecessary hospital admissions of the patients and therefore foster a higher quality of life for patients and their family caregivers as defined by the World Health Organisation [2].

As in other European countries, most patients in Germany (67 to 90%) wish to die at home [3-5]; although, in reality, a hospital is the most common site of death [5-7]. Hospital admissions are common in the last days of life [8,9], often because family caregivers feel unable to cope with the situation and do not receive appropriate professional help [10].

In the UK, care at home is supported by the government [11] since it enables patients to maintain an individual lifestyle and improves their quality of life [12]. Factors influencing the place of care at the end of life include family/social support, the preferences of patients and contacts with health care providers [13]. To receive sufficient support in caring for patients, family caregivers need to know about patients’ symptoms, available information and spiritual support where appropriate [11]. A systematic review summarized studies of interventions with family caregivers to reduce their burden and found that evidence was moderate in dementia and weak in cancer [14]. Furthermore, studies with family caregivers of dementia patients often introduced an additional professional (case manager, coordinator, etc.) for the duration of the study but these were no longer available after the cessation of the study (and the associated funding).

In Germany, the burden of, and relief strategies for, family caregivers has also been studied mainly for caregivers of dementia patients [15-17]. However, the results cannot be transferred easily on to family caregivers of patients with other conditions. Dementia is characterized by long-term and slowly progressing functional disability, whereas health in other diseases, i.e. incurable cancer, declines rapidly [18], posing other challenges on family caregivers. In all diseases, different phases of care are accompanied by different kinds of burden [19,20]. At the time of diagnosis, family caregivers are concerned about the new and challenging situation, understanding the symptoms, and the effects on the patients’, and their own, life. At the same time, they tend to underestimate the burden [20]. Issues can include long-term care; for example limitations on quality of life, financial constraints, the need to give up work, and having to engage with and navigate the health care system. At the end of life, family caregivers struggle with reacting to the worsening health status of patients, and often experience anxiety and depression. Finally, the time of bereavement has its own challenges [20].

General practitioners are perceived as ideal key workers in meeting the support and counselling needs of family caregivers [21]. As such they and their practice teams are expected to identify and offer relief to caregivers as appropriate taking individual characteristics, i.e. gender, age and other obligations, into account [22].

Family caregivers should be informed about the options for burden relief, i.e. about support offered about administrative issues, counselling centres, social services and home assistance services, to identify and address early burden relief and therefore to enable and sustain care at home. Often this information is not forwarded to family caregivers who need to be pro-active to find out about such services [23].

In Germany, the guideline “*Informal caregivers*” was issued by the German Society for General Practice and Family Medicine in 2005 [24]. The authors provide a general information sheet for caregivers, but also make it clear that information about specific regional services within reach of caregivers need to be provided by the practice team. There are no studies examining the implementation of this guideline in practices.

Internationally there is a need for longitudinal studies which involve practice teams, patients and caregivers to support the development of interventions [25] to sustain or improve the quality of life of patients and family caregivers and at preventing or delaying hospital admissions due to overburdened family caregivers [26].

There are international guidelines in structured end-of-life care which are not easily transferable to the German health care system. The Gold Standards Framework (GSF) of the UK, for example, requires a nominated co-ordinator for palliative care within the primary health care team on a basic level of adoption [27]. Primary health care teams in the UK comprise several members with diverse professional backgrounds, i.e. general practitioners (GP), district nurses, managers, practice nurses etc. In Germany, on the other hand, the size of practices is smaller, with about half of general practices single-handed (1 GP) with a small team of doctors’ assistants-comparable to a qualification between that of a health care assistant and practice nurse. Involvement in patient care depends on the individual additional qualifications staff have achieved, and needs a delegation of tasks of the employing GP who holds the final medical responsibility and liability. Meeting tasks stipulated in the GSF focusing on communication and carer support could be adapted only if such co-ordinator role can be identified.

Another important aspect of the German situation is that no financial incentives exist for primary palliative care. However, there is a specialist-driven development for special ambulatory palliative medicine (SAPV). Within SAPV specialists or specialist teams offer support from one-off consultations to full palliative care [28]. This system targets patients with an incurable, progressive and life-limiting illness who need extensive care which cover only approx. 10% of patients who are in a palliative situation [29].

The PalliPA project (Verbesserung der häuslichen Versorgung von Palliativpatienten durch Unterstützung pflegender Angehöriger-Improvement of palliative care at home by supporting family caregivers) focuses on one aspect of end-of-life care important throughout the care process: the role of general practice teams in supporting family caregivers of patients at the end of life. The project is framed as an exploratory trial for a subsequent implementation study.

### Aim and design

In this study, supporting structures and procedures for palliative care patients and their family caregivers will be developed and tested in a sample of general practice teams (GPTs) with a special interest in palliative care. This is based on the premise that those practice teams are more highly motivated to identify a gold standard for palliative care in general practice than a representative sample.

There are two main issues regarding caregivers in general practice which are addressed in the study:

(1) What do GPTs do to identify burdened family caregivers?

(2) How can GPTs support burdened family caregivers? What options do they have to offer burden relief and to where can they refer burdened family caregivers (i.e. offers in the community)?

**Figure 1:**
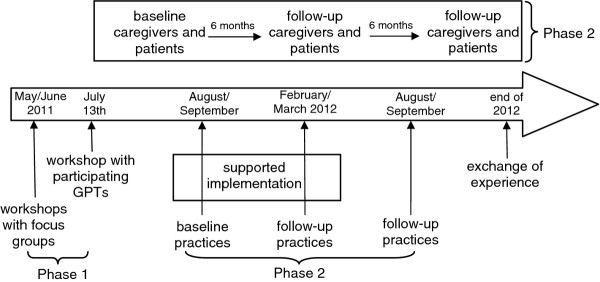
Timeline of the PalliPA study.

To enable an evaluation the intervention needs to be defined sufficiently and developed rigorously. Campbell et al. recommended a concurrent observation of the first 3 phases of the framework for design and evaluation of complex interventions [30]. Phases 0 (preclinical/theory) and I (modelling) are combined with phase II (exploratory trial) to understand the problem, the intervention, and the evaluation [31]. Table 1 and Figure 1 give an overview over the PalliPA study within this framework.

**Table 1:** **Phases of the MRC framework**[27]in the PalliPA study*

	**Current study**			**Planned future work**	
Phase	0	I	II	III	IV
MRC framework description	Preclinical or theoretical (why should this intervention work?)	Modelling (how does it work?)	Exploratory or pilot trial (optimising trial measures)	Definite randomized controlled trial	Implementation
PalliPA study context	Existing evidence: German guideline “Informal caregivers”, results from international implementation and intervention studies, results of caregivers’ needs assessments	Workshops: GPTs are informed about the existing evidence and discuss individual strategies for identifying and relieving burdened caregivers	Participatory action research: individual strategies are implemented in the practices and refined within a Plan-Do-Study-Act cycle	Not part of the PalliPA study	Not part of the PalliPA study
			Estimation of effects on caregivers and patients as a basis for the planning of a subsequent trial		

* The PalliPA study refers to Phases 0-II.

Phase 0 (‘Why should the intervention work’?): Implementation studies in general practices show interventions are best adapted if they can easily be integrated into existing practice procedures, GPTs are motivated and committed to the implementation, they receive support and the implementation is evaluated [32]. The PalliPA project is based on the Scottish Action Research study for the improvement of cancer patients’ and their family caregivers’ care [32], the guideline “Informal caregivers” of the German Society for General Practice and Family Medicine (DEGAM) [24] and results of needs assessments [11,15,20,25]. GPTs take part in developing and implementing interventions. The feasibility and practicability of the intervention will be evaluated. GPTs use existing structures and procedures and optimize them together with a supporting research team. Involving the GPTs leads to an early recognition of burden in family caregivers with the chance to offer relief before the burden develops into a serious problem.

Phase I (‘How does the intervention work’?): In workshops, needs and resources of family caregivers are discussed with GPTs with special emphasis on stages of care [20] and the socio-demographic background of caregivers (employment status, age, gender, own disabilities, other persons to care for). Additionally, the DEGAM guideline [24] provides information to recognize caregiver burden which has to be contextualised to end-of-life care.

### PalliPA-Qualitative

#### Data assessment

As part of the workshops in phase I, facilitators and barriers to good palliative care in general practice were discussed in focus groups with the GPTs. The focus groups were digitally recorded and transcribed verbatim for analysis.

#### Sample and sample size

The sample consisted of GPTs from the federal state of Baden-Wuerttemberg. GPTs had to currently take care of palliative patients. Each GP with an additional qualification in palliative medicine listed in the directory of the Association of Statutory Health Insurance Physicians (Kassenärztliche Vereinigung) was invited to participate. Invitations were mailed to GPTs in March 2011.

We planned 3 workshops with 5 GPTs each. We conducted 2 workshops in May and June 2011, where information on the project was given and the current support provided for caregivers was discussed in focus groups. 19 GPs and doctor’s assistants participated in 4 focus groups. In addition, 2 GPs were interviewed via telephone since they were not able to participate in the workshops.

#### Data analysis and implication of results

Transcripts of the focus groups are being analysed using qualitative content analysis [33]. Qualitative content analysis means an inductive development of categories and a deductive application of categories, where key issues are identified, summarized, labelled as codes and sorted into main and sub-categories. Primarily, improvement strategies to relief caregiver burden as proposed by the GPTs were extracted from the transcripts. These were complemented by results from the literature to create a comprehensive list of potential interventions in general practice. In a second meeting with the GPTs in July 2011, as part of the iterative process, preliminary findings of the focus group were presented and discussed. Based on these findings the development of a first set of tools to be used in the practices was discussed and agreed upon. Immediately after this meeting, GPTs started to include patients and caregivers in a subsequent exploratory trial.

In implementation, both the services provided by practices and the circumstances of patients and caregivers need to be considered alongside the improvement strategies mentioned in the focus groups. In previous studies, various facilitators or barriers in the practices were identified, i.e. the absence of a nominated contact person for caregivers or a lack of cooperation with a nursing service [34]. Supporting or inhibiting factors associated with caregivers were also identified, i.e. good experiences with a nursing service [35] or dealing with guilt when taking respite from patient care [36]. Recognising and addressing such explicit and implicit resources and barriers are important aspects of preparing and successfully implementing an intervention [37].

Phase II: In the exploratory trial following the focus groups interventions are constantly evaluated and refined within a participatory action research approach [38] to create a set of relevant, acceptable and feasible tools to implement in general practice. The participatory action research approach enables the GPTs to take an active part in the development of the implementation. Smaller changes of one’s own choice have a greater chance of being accomplished than regarding all problems at once. GPTs can observe changes more easily and are motivated to try new solutions, to adapt them to their practice routine [25,32] and to maintain them. GPTs are able to immediately and flexibly react to the implementation into their existing practice procedures, thus fostering their motivation to participate and to develop feasible tools [32].

In September 2011, the Phase I first set of tools was sent to the GPTs. As a result from the meeting in July and reports in the literature, it comprised a practice register of palliative care patients, a master data sheet for each patient including information on caregivers, a summary sheet of issues for detecting burden in caregivers, and a leaflet with local addresses offering support for patients and caregivers.

Ten GPTs are participating in this phase of implementation from September 2011 to June 2012 (Figure 1). Each GPT develops its own individual strategy for identifying and relieving burdened caregivers. For 6 months (09/2011 to 03/2012) members of a scientific team from the University Hospital Heidelberg will support GPTs by visiting the practices, by discussing implementation and occurring problems with the GPTs and by adapting burden relief strategies as necessary. GPTs regularly feed back on the tools aiming to improve them for wide-spread use. From April 2012 to June 2012 GPTs will then be supported only via telephone and only visited as requested. Another half year later (12/2012) (without further feedback), the implications of the project are reflected upon in another focus group which will discuss: the overall experiences within the project; which tools are still in use in the practices; and whether patients and/or caregivers gave any informal feedback regarding their support.

### PalliPA-Quantitative

#### Data assessment

Accompanying the intervention, a sample of patients and family caregivers evaluate their quality of life, family caregiver burden and healthcare costs during development (baseline) and implementation (6-month follow-up and, where possible, 12-month follow-up for long-time effects). Patients and caregivers are included in the study between July 2011 and April 2012, with follow-ups planned between January and October 2012 and July 2012 and April 2013.

Caregiver burden is assessed by the German version of the Burden Scale for Family Caregivers (BSFC, Grässel et al. 2003 [39]). Quality of life is assessed by the German version of the Quality of Life Questionnaire Core-15 Palliative Care (QLQ-C15-PAL [40]) of the European Organisation for Research and Treatment of Cancer. Health care expenditures are assessed using a standardized questionnaire. All parameters are used for an estimation of possible effects over time with which the sample size of a future controlled study can be derived.

In the practices direct effects on the practice structure are measured as well, i.e. the existence of an up-to-date register with patients and their caregivers, the offer of home visits to patients and caregivers, and if the practice informs burdened caregivers about relief options offered by the community.

Based on the results of both feedback from the GPTs as well as of the patient and caregiver survey a controlled implementation study will be planned after the completion of PalliPA in December 2013 (Table 1).

#### Control group

The quantitative results will be used for estimating the effects of the intervention on patients and family caregivers prerequisite for planning and conducting a randomized controlled trial. Because of the longitudinal design of the PalliPA Quantitative we will be able to refer to the results of a historical control group to put the results into a wider context. The evaluation of PAMINO (PAlliativMedizinische Initiative NOrdbaden) [41] was conducted between September 2007 and June 2009 with patients with advanced cancer and their family caregivers in general practices. The questionnaires QLQ-C15-PAL and the short version of the BSFC were applied, which are also used in the PalliPA project.

### Sample and sample size

Participatory action research approaches, combined with an exploratory trial, places high demands on participants. Therefore, in PalliPA, 10 practice teams are developing, implementing and evaluating support tools in their practice. In each practice, 4 patients with their family caregivers are included in the exploratory trial (07/2011 to 12/2012) to estimate effects on patients’ quality of life and family caregiver burden (Figure 1).

Patient-caregiver dyads are informed about the exploratory trial by the GP or the doctor’s assistant. GPTs include adult outpatients (at least 18 years of age) with an incurable and life-threatening illness (i.e. cancer, heart failure, chronic obstructive lung disease, post stroke) and a family caregiver who mainly supports the patient. Both have to give their informed and written consent to participate in a questionnaire study.

Patients or caregivers suffering from dementia are not included in the study, since they are not able to consent to study participation at a later disease stage and cannot give information about their quality of life (by questionnaires). Furthermore, there are comprehensive studies in supporting caregivers of dementia patients [17]. Insufficient German language skills in patients and caregivers also lead to exclusion from the study.

We need a sample size of at least n = 30 patient-caregiver dyads to be able to estimate effects from an exploratory trial [42]. Based on our experiences with the PAMINO evaluation we assume 1 or 2 eligible cancer patients cared for in a practice during the implementation period. Cancer patients comprise about 25–30% of all palliative care patients in general practice in Germany [43,44]. Therefore, we could assume a further 2 to 4 patients per practice may be recruited. For a sufficient data base of 30 patients and caregivers at least 40 dyads should be enrolled in the study to compensate for attrition and missing data. Based on these assumptions we need to enrol 8 to 10 GPTs with 4 to 5 patient-caregiver dyads each in the exploratory trial.

### Statistical analysis

The primary outcome of the exploratory trial is the effect of the implemented interventions on caregiver burden. Results on the BSFC will be compared after the 3 assessments (baseline and 2 follow-ups) using ANOVA (analysis of variance) with repeated measures if data are normally distributed. Otherwise, the Friedman test will be used. Secondary outcomes on the quality of life of patients will also be analyzed using ANOVA with repeated measures or Friedman test, respectively. Data of practice structure will be described as frequencies and analyzed with the Cochran test. Health care costs will be described cross-sectionally and longitudinally. The assessment will show how feasibly these parameters can be measured in general as well as a first estimate of expenditure.

## Discussion

Quality of care is at its most meaningful when related to meeting the needs of individual patients [45]. In no context is this more important than during the period of palliative care. The care of patients with a life-threatening, progressive and end-of-life illness in a home-care setting requires appropriate individual patient care and the active support of family caregivers. However, the support given to carers is an under-researched area [25]. The PalliPA project focuses on the role and options of general practice teams in supporting family caregivers of patients at the end of life in Germany. While there are international guidelines related to end-of-life care, including the UK Gold Standards Framework [27], these are not easily, and should not be automatically, transferable to the German health care system as health care systems differ substantially regarding many structural and organisational aspects. Within the stepwise approach of the PalliPA project, general practice teams will be supported in the active development of a feasible, acceptable and successful strategy to support family caregivers of patients at the end of life. In addition, the project is framed as an exploratory trial for a subsequent implementation study, covering phases 0, I and II of the MRC (Medical Research Council) framework [31] for development, design and evaluation of complex interventions. The strategies developed within the project will be appropriate for primary palliative care in the German health care context and best practice end-of-life care.

### Ethics

The study is being conducted in accordance with the Medical Professional Codex of the state of Baden-Wuerttemberg (2007) and the Helsinki Declaration as of 2008.

The study protocol was approved by the ethics committee of the University of Heidelberg in May 2011 (approval number S-042/2011) with an unrestricted positive vote.

## Abbreviations

ANOVA: Analysis of Variance; BSFC: Burden Scale for Family Caregivers; DEGAM: Deutsche Gesellschaft für Allgemeinmedizin und Familienmedizin (German Society for General Practice and Family Medicine); GP: General practitioner; GPT: General practice team; GSF: Gold Standards Framework; MRC: Medical Research Council; PalliPA: Verbesserung der häuslichen Versorgung von Palliativpatienten durch Unterstützung pflegender Angehöriger (Improvement of palliative care at home by supporting family caregivers); PAMINO: Palliativmedizinische Initiative Nordbaden; QLQ-C15-PAL: Quality of Life Questionnaire Core 15 Palliative; SAPV: Spezialisierte Ambulante Palliativversorgung (Special ambulatory palliative medicine).

## Competing interests

The authors declare that they have no competing interests.

## Authors’ contributions

KH is the principal investigator of the study and wrote the first draft of the manuscript. All authors were involved in the planning of the study and have read and approved the final version of the manuscript.

## Authors’ information

RB, PE and FPK are practicing general practitioners with an additional qualification in palliative medicine.
